# Prevalence of Chikungunya, Dengue, and West Nile arboviruses in Iran based on enzyme-linked immunosorbent assay (ELISA): A systematic review and meta-analysis

**DOI:** 10.1016/j.gloepi.2025.100202

**Published:** 2025-04-28

**Authors:** Ebrahim Abbasi, Mohammad Djaefar Moemenbellah-Fard

**Affiliations:** a Research Center for Health Sciences, Institute of Health, Shiraz University of Medical Sciences, Shiraz, Iran; b Department of Biology and Control of Disease Vectors, School of Health, Shiraz University of Medical Sciences, Shiraz, Iran

**Keywords:** Arbovirus, Chikungunya, Dengue, ELISA, Meta-analysis, Virus, WNV

## Abstract

**Introduction:**

Arboviruses, including Chikungunya (CHIKV), Dengue (DENV), and West Nile (WNV) viruses, are significant viral threats that affect numerous people globally each year. This report explores the prevalence of these viruses in Iran through a systematic review and meta-analysis.

**Methods:**

The present survey was performed using a systematic review and meta-analysis method on the seroprevalence of WNV, CHIKV, and DENV using the ELISA test. Accordingly, by searching Web of Science, PubMed, Scopus, Cochrane Library, Science Direct, and Google Scholar scientific databases, all relevant published papers were sorted out and reviewed. Power ratification of data was conducted with a random effects model in meta-analysis, meta-regression, *I*^*2*^ index, and Egger test.

**Results:**

This meta-analysis report embodies twelve published papers between 2000 and 2024. The seroprevalence of positive ELISA tests for WNV in Iran was estimated at 12.9 % (CI = 95 %: 7.4–18.4) and for CHIKV at 6.2 % (CI = 95 %: 0.6–11.8). Regarding DENV, only two studies were conducted, with a zero prevalence in one study and a seroprevalence of 5.6 % in another study.

**Conclusion:**

According to these data, WNV, CHIKV, and DENV fevers have been detected in Iran using the ELISA test. Considering the seropositivity of WNV and CHIKV and their detection in several provinces, it can be assumed that these viruses are ubiquitous, while DENV fever remains sporadic in Iran.

## Highlights

• This study highlights the prevalence of three significant arboviruses—West Nile Virus (WNV), Chikungunya Virus (CHIKV), and Dengue Virus (DENV)—in Iran through a systematic review and meta-analysis.
• The analysis, based on 12 studies conducted between 2000 and 2024, revealed that the seroprevalence of WNV was 12.9 %, and CHIKV was 6.2 %, indicating these viruses are widespread across multiple provinces in Iran.
• In contrast, DENV was found to be sporadic, with only one study showing a seroprevalence of 5.6 %.
• These findings underscore the presence and potential public health impact of these arboviruses in the region.

## Graphical abstract

**Figure:**
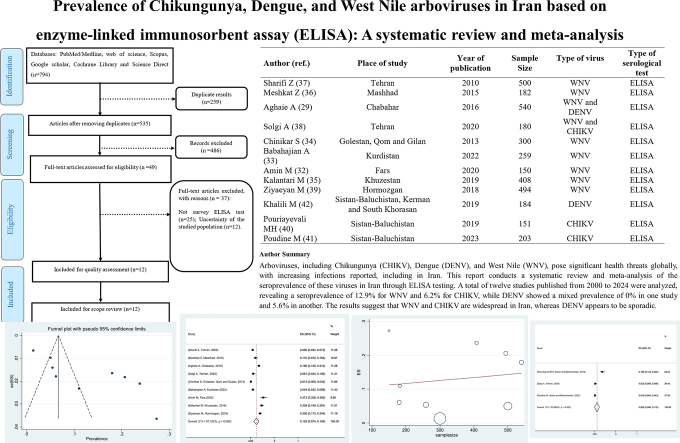

## Introduction

West Nile virus (WNV) is a single-stranded positive-sense RNA virus in the Flaviviridae family, primarily transmitted by *Culex* and *Aedes* mosquitoes. *Culex pipiens*, *Culex tarsalis,* and *Aedes vexans* are the main vectors of WNV [1,2]. In Iran, *Cx. pipiens* and *Ae. caspius* are vectors of WNV. *Trans*-ovarian transmission of WNV among mosquito vectors is also possible, which renders them reservoirs of this pathogen. WNV circulates between insects, birds, and mammals (as the dead-end host), and their life is maintained in a cycle of “amplification” [[2], [3], [4]]. The virus cannot be transmitted directly between mammals, including human-to-human transmission. It can only spread to another person through organ transplants, blood transfusions, or placental and milk transmission. Accidental human infection is often (≈80 %) asymptomatic, in 20 % of cases with mild flu-like symptoms, and < 1 % can cause WNV neurotropic disease (WNND, West Nile Neuro-invasive Disease), which usually occurs in elderly and immunocompromised individuals, that can lead to death.

Migratory birds play significant roles in the transmission and spread of WNV, and this virus has recently become prevalent in most parts of the world, including Europe and Asia. Since no effective and safe vaccine is available to deal with this virus [9], organizations and departments related to health have suggested that WNV monitoring plans be implemented in the form of collecting virologic, entomologic, veterinary, and epidemiologic data to monitor the spread of WNV and identify cases of human infection so that prevention, control, and adoption of appropriate treatment protocols to deal with this virus could be undertaken.

Dengue virus (DENV) is also a single-stranded positive-sense RNA virus in the Flaviviridae family and the causative agent of the newly emergent dengue fever, which has four serotypes of 1, 2, 3, and 4 [13]. The primary and secondary vectors of DENV are *Aedes aegypti* and *Aedes albopictus* mosquitoes [14,15]. *trans*-Ovarian transmission of DENV among vectors is also possible. The life of all four DENV serotypes is maintained in two sylvatic and urban cycles. The Asian tiger mosquito, *Ae. albopictus*, serves as a “bridge” vector between these two cycles. Both these diurnally-active anthropophilic *Aedes* vectors are increasingly reported from SE (Southeast) Iran. In the sylvatic cycle, the virus is transmitted from non-human mammals to mosquitoes and then to mammals, such as the monkey-*Aedes*-monkey cycle, which is ecologically and evolutionarily distinct from the human transmission cycle [16].

More than 400 million infections, and > 20,000 deaths occur annually due to DENV worldwide [13,17]. This disease often exists as a latent or asymptomatic infection and in endemic/epidemic forms in different regions of the world [13]. Low-to-medium viremia in these individuals could be infectious to mosquitoes, contributing to the annual DENV epidemics observed across Asia, America, Africa, and Australia [18]. Clinical manifestations of dengue infection range from mild fever to severe dengue hemorrhagic fever (DHF). Infection with one serotype of DENV can provide lifelong immunity to the same serotype, while secondary infection with other serotypes levitates the risk of infection to severe DENV. No effective vaccine is available for this virus [19].

Chikungunya virus (CHIKV) is a single-stranded positive-sense RNA virus of the alphavirus genus in the Togaviridae family. Alphaviruses can cause inflammatory musculoskeletal diseases with debilitating symptoms such as arthritis, arthralgia, and myalgia in humans [20]. CHIKV is transmitted by *Aedes* mosquitoes, especially *furcifer*, *africanus*, *aegypti*, *albopictus*, and *Stegomyia* species [21,22]. This virus has three genotypes, namely West African (WA), East/Central/Southern African (ECSA), and Asian. All three genotypes are distributed globally, but ECSA and Asian genotypes are more common. The virus is maintained in a rural enzootic transmission cycle or the sylvatic cycle between *Aedes* mosquitoes and animal reservoirs. However, the virus has lately adapted to urban cycles and no longer requires the presence of non-human primates or the sylvatic cycle for their maintenance [25].

Unlike DENV, CHIKV is not life-threatening. Mortality caused by this virus is low, but it can cause severe complications, affecting people's quality of life. In most cases, CHIKV infection starts with a sudden onset of fever accompanied by joint pain. In minor cases, it leads to polyarthralgia and debilitating arthritis, rashes, myalgia, and headache [26]. On the other hand, asymptomatic infection is rare and occurs in 3–28 % of cases in epidemics [27,28]. The infection is often self-limiting, and the patient eventually recovers. However, some patients develop persistent joint pain that may last for months or years after the acute stage of the disease. Approximately 30–40 % of infected individuals experience some long-term complications [31]. Currently, no vaccine has been developed to protect against this virus [21].

Chikungunya virus (CHIKV) was previously shown to cause mild infection, while evidence increasingly emphasizes its significant public health burden due to persistent and chronic effects. CHIKV infections are often accompanied by severe musculoskeletal complications, including chronic polyarthritis and arthralgia, which can persist for months or even years. Moreover, viral persistence in tissues has been documented, contributing to long-term complications [29,31]. Beyond musculoskeletal issues, pre-natal exposure to CHIKV has been associated with adverse birth outcomes, including microcephaly and developmental delays. These findings elevate CHIKV from a mere febrile illness to a substantial public health concern requiring robust surveillance and control strategies. Importantly, the FDA's approval in November 2023 for clinical trials of a new CHIKV vaccine marks a critical step toward mitigating this disease's impact.

The ELISA (Enzyme-Linked Immunosorbent Assay) test is a widely used method for the serological examination of viruses worldwide. Its high sensitivity, coupled with a low false positive rate of approximately 20 %, makes it a recommended choice for the initial screening of viral diseases. The presence of DENV IgG antibodies in people indicates that they have been infected with the virus in the past or present, which is usually investigated in the ELISA test [33].

However, like any serological assay, ELISA has some limitations that warrant careful consideration. Cross-reactivity is a notable concern, particularly in regions where multiple arboviruses co-circulate, as antibodies targeting one virus may exhibit binding affinity to antigens of a different virus, potentially leading to false-positive results [34]. This issue is particularly critical for flaviviruses such as Dengue and West Nile, sharing antigenic similarities. Additionally, ELISA's performance heavily depends on rigorous standardization of reagents and protocols to ensure reproducibility and minimize variability across studies. Addressing these caveats through assay validation and the inclusion of confirmatory molecular diagnostics, such as RT-PCR, enhances the reliability of seroprevalence estimates obtained via ELISA.

Due to the lack of an effective vaccine to prevent viral infections of arboviruses and the existence of complications and death caused by these viruses, awareness of the prevalence, distribution, and detection methods of these viruses is essential to adopt control and prevention programs. The viral diseases mentioned have been recognized in Iran in recent years. However, their prevalence is different in different regions of Iran. Accordingly, this study was conducted to determine the positive rate of ELISA tests for WNV, DENV, and CHIKV using a systematic review and meta-analysis method in Iran to achieve comprehensive results regarding the spread of these viruses.

## Materials and methods

### Study protocol

This study was conducted using systematic review and meta-analysis regarding ELISA tests used for the three arboviruses of WNV, CHIKV, and DENV, based on the guidelines of PRISMA (Preferred Reporting Items for Systematic Reviews and Meta-Analyses) [38].

### Search strategy

In the initial search, all English-language articles published from the beginning of 2000 to the end of May 2024 were extracted by searching the Web of Science, PubMed, Scopus, Cochrane Library, Science Direct, and Google Scholar databases. Using the keywords of Arbovirus, West Nile Virus, West Nile Virus Infection, WNV, West Nile Virus IgG, IgG anti-WNV, Dengue Fever, Dengue virus, Dengue virus infection, Chikungunya virus, Enzyme-linked immunosorbent assay, ELISA, Serology, Seroprevalence, Seropositivity, Iran, Iranian, the title, abstract and keywords of the collected articles were searched in singular and compound form using “AND” and “OR” operators.

### Inclusion and exclusion criteria

All English-language articles published in Iran on arboviruses, namely WNV, CHIKV, and DENV, were included in the study. These studies used the ELISA test for serological examination, and those reporting a positive rate of good quality were selected for analysis. Articles that did not meet the inclusion criteria or were conducted using meta-analysis, review, case report, or case series methods were excluded from the study.

### Quality assessment

The quality assessment of the articles was done based on 22 parts of the STROBE (Strengthening the Reporting of Observational Studies in Epidemiology) checklist, which examined compliance with the principles of writing and implementation in the title, the method of reporting findings, limitations, and conclusions. Each part of this checklist is given a score based on its importance, and the maximum possible score is 33 [39].

### Screening and data extraction

The articles were independently screened by two researchers based on titles, abstracts, and inclusion/exclusion criteria. The full texts were then reviewed independently by both researchers, with any disagreements resolved by a third reviewer. Articles rejected by both researchers were excluded with a provided rationale. Data extraction was done using a pre-prepared checklist that included study location, study time, sample size, type of serological test, number of positive ELISA tests, and type of virus under investigation.

### Study selection

**Fig. 1:**
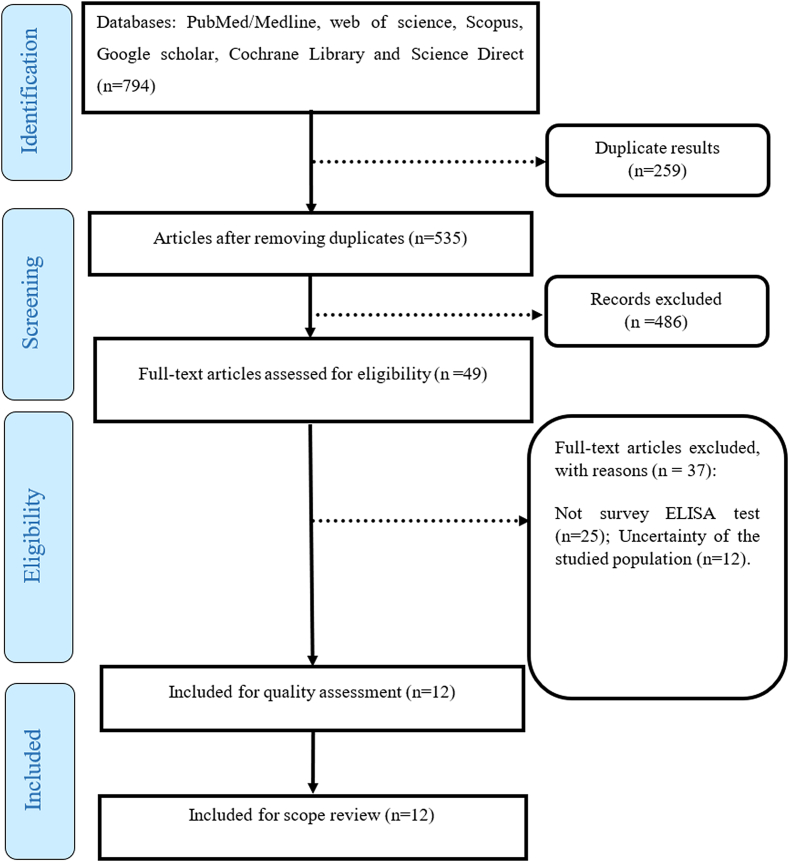
The review process based on PRISMA flow chart.

In the initial search and considering the inclusion criteria, 794 articles were extracted. Then, using the Endnote software, the duplication of sources was investigated, and 259 articles were excluded from the study due to duplication. Subsequently, by investigating the titles and summaries of the articles, 486 articles were excluded from the study due to their irrelevance. After reviewing the full text of the articles, 37 articles were excluded due to the lack of investigation on the prevalence of the ELISA test and the unknown population. Finally, 12 articles entered the meta-analysis process (Fig. 1).

### Statistical analysis

In heterogeneous studies, the random-effects model was used to combine the results, and in inhomogeneous studies, the fixed-effects model in the meta-analysis was deployed. To investigate the heterogeneity of the data, *I*^*2*^ and Cochrane Q tests were used. The Egger test controlled publication bias, and funnel plots and data analysis were performed using STATA version 17.0 software.

## Results

Overall, this meta-analysis included 12 articles conducted in Iran between January 2010 and December 2023. Nine articles on WNV, three articles on CHIKV, and two articles on DENV [33,50] were scrutinized. Table 1 presents the characteristics of the articles included in the meta-analysis.

**Table 1:** Characteristics of the articles included in the meta-analysis.

Author (ref.)	Place of study	Year of publication	Sample Size	Type of virus	Type of serological test
Sharifi Z [45]	Tehran	2010	500	WNV	ELISA
Meshkat Z [44]	Mashhad	2015	182	WNV	ELISA
Aghaie A [33]	Chabahar	2016	540	WNV and DENV	ELISA
Solgi A [46]	Tehran	2020	180	WNV and CHIKV	ELISA
Chinikar S [42]	Golestan, Qom and Gilan	2013	300	WNV	ELISA
Babahajian A [41]	Kurdistan	2022	259	WNV	ELISA
Amin M [40]	Fars	2020	150	WNV	ELISA
Kalantari M [43]	Khuzestan	2019	408	WNV	ELISA
Ziyaeyan M [47]	Hormozgan	2018	494	WNV	ELISA
Khalili M [50]	Sistan-Baluchistan, Kerman and South Khorasan	2019	184	DENV	ELISA
Pouriayevali MH [48]	Sistan-Baluchistan	2019	151	CHIKV	ELISA
Poudine M [49]	Sistan-Baluchistan	2023	203	CHIKV	ELISA

**Fig. 2:**
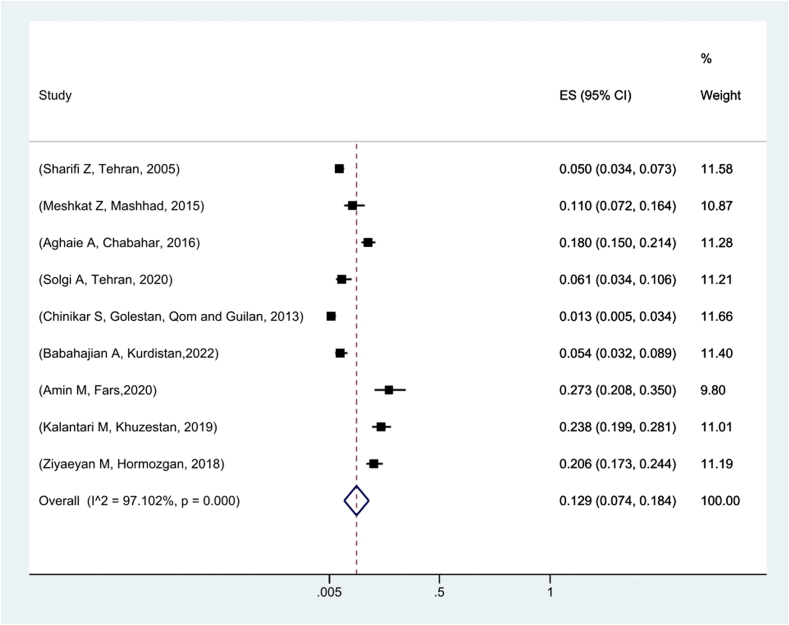
Pooled prevalence of positive ELISA test for West Nile virus in Iran based on the random effects model. The midpoint of each line segment shows the prevalence estimate, the length of the line segment indicates the 95 % confidence interval in each study, and the diamond mark illustrates the pooled prevalence.

Nine articles with a sample size of 3013 people in the field of WNV serology in Iran were included in the meta-analysis process. Based on these findings, the prevalence of WNV was estimated to be 12.9 % using the ELISA serology test in Iran. Among the surveyed studies, the highest prevalence of WNV-positive tests was related to that conducted in Fars Province, with a prevalence of 27.3 %, and the lowest prevalence of the test was related to the studies carried out in Golestan, Gilan, and Qom Provinces with a prevalence of 1.3 % (Fig. 2).

**Fig. 3:**
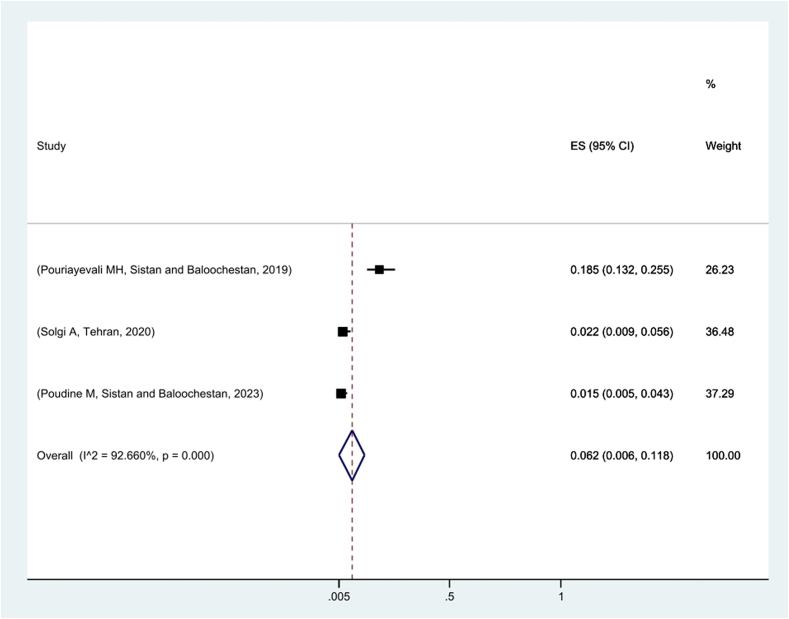
Pooled prevalence of positive ELISA test for Chikungunya virus in Iran based on random effects model. The midpoint of each line segment shows the prevalence estimate, the length of the line segment indicates the 95 % confidence interval in each study, and the diamond mark illustrates the pooled prevalence.

The meta-analysis of three studies performed on the prevalence of positive CHIKV assay in Iran showed that 6.2 % of the studied population had a positive ELISA test in this regard (Fig. 3). The prevalence of positive Dengue fever virus ELISA test in Iran was also examined in two studies. In one survey [50], there was no case of positive ELISA test in the field of DENV, while the prevalence of this virus was reported at 5.6 % in the other study conducted in Sistan-Baluchistan Province.

**Fig. 4:**
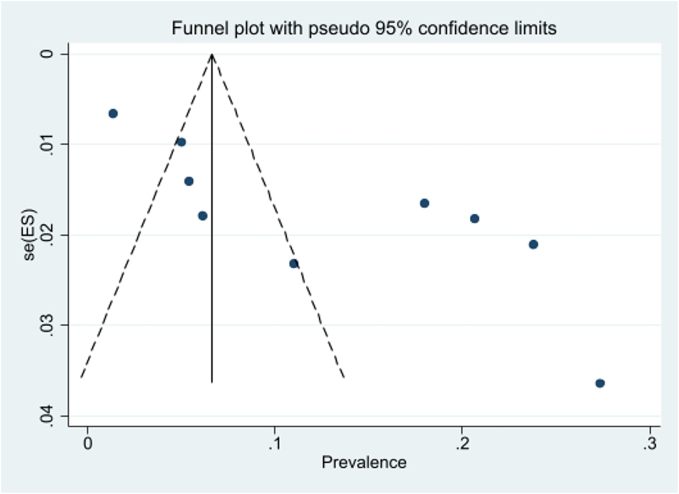
Funnel plot of the prevalence positive ELISA test in the selected studies.

**Fig. 5:**
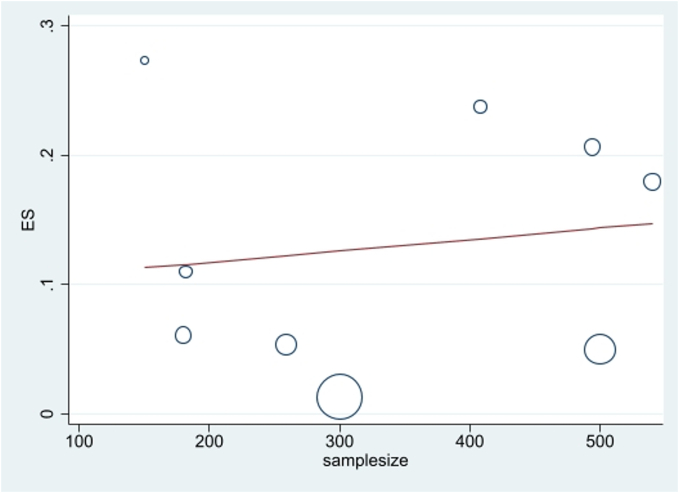
Meta-regression plot of prevalence of West Nile virus and sample size of study.

The publication bias was evaluated using a funnel plot and Egger test. Considering the symmetry of the funnel plot and the fact that the studies with a high sample size are placed under the plot, it can be mentioned that the publication bias did not occur (*P* = 0.12) (Fig. 4). Although several studies with smaller sample sizes fall outside the graph, the findings highlight the need for additional studies in this field to establish a consensus. Investigating the prevalence of positive ELISA tests based on the current sample volumes showed that with the increase in sample size, the prevalence of positive ELISA tests has also increased (Fig. 5).

## Discussion

Based on the findings of this meta-analysis concerning the use of the ELISA serology test in Iran, the WNV prevalence was estimated to be 12.9 % (95 %, CI: 18.4–7.4). In the past decades, WNV has spread in most regions of the world, which has affected its virulence, pathogenicity, epidemiology, and hosts. WNV has been responsible for major epidemics worldwide, resulting in thousands of cases of human morbidity and mortality [52]. This virus is endemic in the Middle East, in the countries of Pakistan, Jordan, Turkey, Iraq, Oman, Saudi Arabia, Sudan, Yemen, Egypt and Afghanistan [[53], [54], [55]]. The prevalence of WNV was 26.6 % in Pakistan, 30.4 % in Afghanistan, and 10.4 % in Qatar. Its prevalence has also been reported in Iraq (11.6–15.1 %), Egypt (1–61 %), Jordan (8 %), Iran (0–30 %), Libya (2.3 %), Lebanon (0.5–1 %), Pakistan (0.6–65 %), Morocco (0–18.8 %), Tunisia (4.3–31.1 %), and Sudan (2.2–47 %) [58].

WNV is ubiquitously endemic in Iran, with its prevalence varying across regions. Factors such as weather conditions, including temperature and fluctuations in relative humidity, significantly influence the activity of the virus's vectors [59]. In general, the prevalence of WNV in Iran is close to the endemic areas and neighboring countries of Iran. According to the results of surveys included in this meta-analysis, the prevalence of this virus in the southern provinces was higher than in the northern and western provinces of Iran, which can be related to the increased amplification of its virus vectors in the south and neighboring countries of Iran, which showed a higher prevalence of this virus. In general, the prevalence of WNV in Iran is relatively high, and it is crucial to evaluate the circulation of this virus annually, and implement preventive measures to mitigate the spread of this virus in the country.

The results of a meta-analysis on the prevalence of positive ELISA tests for CHIKV showed that this virus prevalence was 6.2 % (95 %, CI: 0.6–11.8) in Iran. Studies have shown that the prevalence of CHIKV varies in different regions of the world. Its epidemics occur every 7–8-year period. The CHIKV is not life-threatening, unlike DENV. The prevalence of CHIKV has been reported in Italy as 10.2 % [66], in India as 22.3 % [67], in Tanzania as 3.7 % [68] and in Turkey as 0.4 % [69]. Also, the presence of CHIKV has been reported solely in Saudi Arabia, Pakistan, Sudan, Yemen, Somalia, Egypt, Oman, Iraq, and Kuwait. It is known to be endemic in many parts of these regions [10,70]. In general, it can be noted that this disease has a relatively high prevalence in Iran, and Iran can be considered an endemic region for CHIKV. Although this disease causes more complications for people, and its attenuation is relatively low, having sufficient information about its prevalence is essential to designing control programs. Based on this, it is necessary to assess and monitor the serological prevalence of this virus annually.

Regarding the Dengue fever virus (DFV), only one study was conducted in Sistan-Baluchistan Province, in which the prevalence of this virus was found to be 5.6 %. Other studies were conducted in the form of reports of Dengue fever in Tehran (2012 and 2009) [71,72], and it showed that this virus was detected in different years in Iran. However, apart from the SE regions of Iran, it has occurred sporadically in other regions. It should also be noted that most of these cases were often reported as imported cases.

Dengue fever is recognized as a disease in developing countries of SE Asia, and most cases occur in these countries [73]. Pakistan, Yemen, Saudi Arabia, Madagascar, and Sudan are among the countries where dengue fever is reported as endemic [74]. In India, the prevalence of dengue fever is 23 %, and in Sudan, it is 47.6 % [75,76]. These countries and SE Asian countries are often considered tourist destinations for Iranians, and many people from Iran travel to these countries every year. As a result, the possibility of contracting DFV and transferring it inside the country increases, especially among travelers returning from Saudi Arabia following the pilgrimage [71,77]. In general, it can be mentioned that cases identified in other provinces of Iran, except Sistan-Baluchistan Province, can be imported cases from other countries and often occur sporadically.

Approximately, 3.9 billion people in 129 countries are at risk of contracting DENV worldwide. Almost 70 % of this global burden of DENV is related to Asia. According to the World Health Organization (WHO), DENV cases have levitated more than eight times in the last two decades. Several important outbreaks of DENV have recently happened in the Eastern Mediterranean Regional Organization (EMRO) countries, including Saudi Arabia, Yemen, Oman, Sudan, and Pakistan. Alarmingly, asymptomatic humans, despite low-medium levels of viremia, transmit DENV to vector mosquitoes. The inter-epidemic period for DENV is 3–5 years.

Additionally, the main anthropophilic vector, *Aedes aegypti*, of DENV is a diurnally-active endophilic species. After seven decades, this mosquito species has re-emerged, particularly in the coastal sea regions of Iran. There are also unconfirmed reports of *Aedes albopictus* presence in the same oriental provinces of this country. Besides, pregnant females and immunocompromised individuals, like those with diabetes, allergies, and many chronic diseases, are especially at risk of being afflicted with DENV.

## Conclusions

Based on the present systematic review and meta-analysis findings, WNV, CHIKV, and DENV have been detected in Iran using the ELISA test. Considering the prevalence of WNV and CHIKV, as well as the identification of these viruses in several provinces, it could be postulated that these two viruses are endemic in Iran, while DENV occurs sporadically in Iran. Accordingly, to monitor and surveil the spread and outbreak of these viruses, it is recommended to screen suspected travelers and high-risk cohorts from highly endemic neighborhood regions using the ELISA test combined with more sophisticated molecular tools such as polymerase chain reactions (PCR).

(*N.B.* While this MS was under the submission process, three new fatal cases of DENV were reported from the SE counties of Fars and Hormozgan Provinces, Iran).

## CRediT authorship contribution statement

**Ebrahim Abbasi:** Writing – review & editing, Writing – original draft, Visualization, Validation, Methodology, Investigation, Funding acquisition, Formal analysis, Data curation. **Mohammad Djaefar Moemenbellah-Fard:** Supervision, Software, Resources, Project administration, Conceptualization.

## Ethical approval

Not applicable. No ethical approval is applicable since it is a review article.

## Funding

No funding was received for this manuscript.

## Declaration of competing interest

The authors declare no conflict of interests.

## Data Availability

The data that support the findings of this study are available from the corresponding author upon reasonable request.

## Appendix A Supplementary data

PRISMA_2020_checklist AND syntax Image 1
